# Supplementary figures and images for: Structure Evolution of Graphene Oxide during Thermally Driven Phase Transformation: Is the Oxygen Content Really Preserved?
Source: PLoS One. 2014 Nov 5;9(11):e111908. doi: 10.1371/journal.pone.0111908 (PMC4221183; doi:10.1371/journal.pone.0111908)

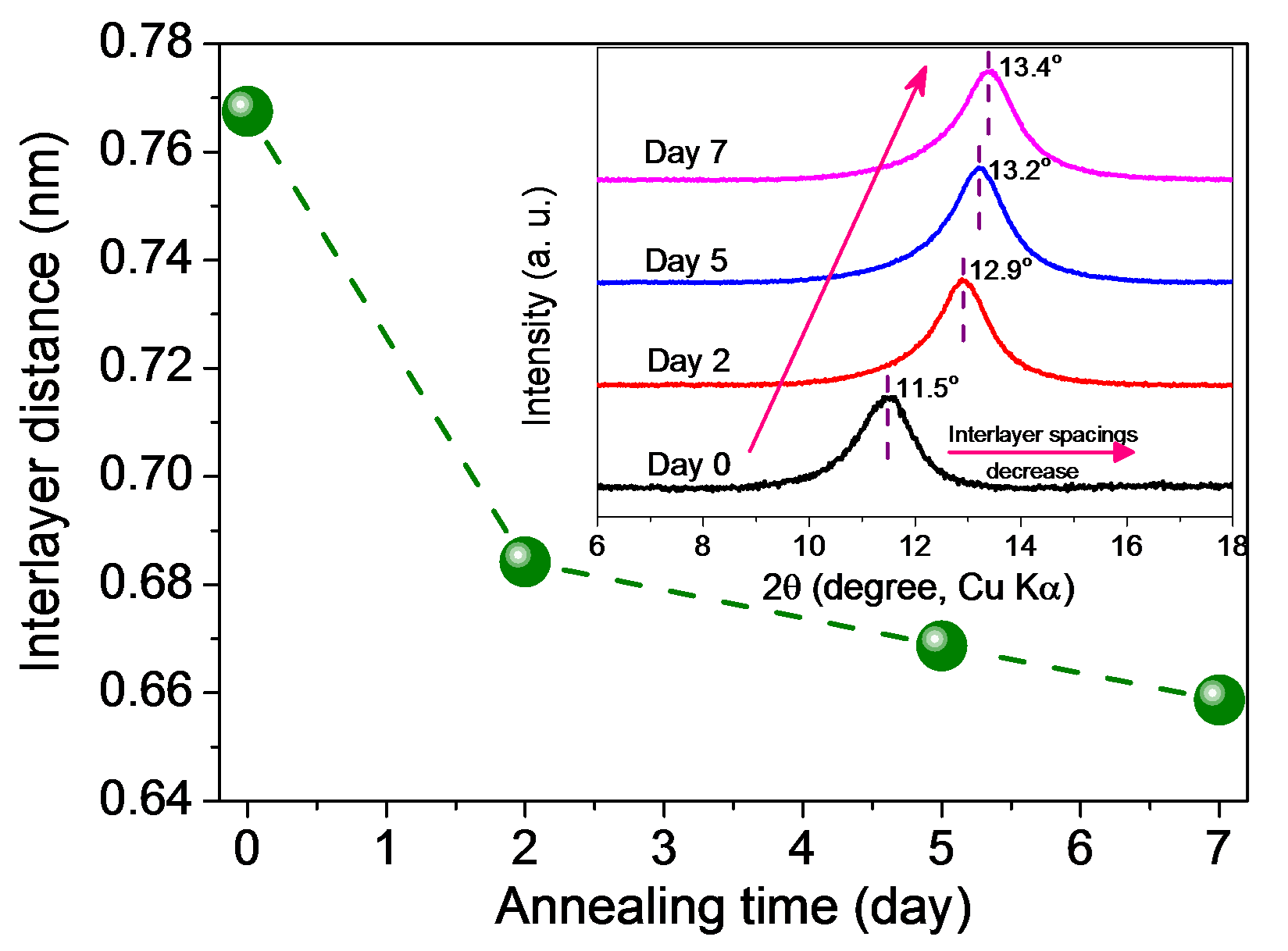

Supplement: Figure S1 — XRD diffractograms of GO laminates during the low-temperature annealing process showing the changes of interlayer distances. (TIF) [file pone.0111908.s001.tif]
